# Long-Term Outcomes of Successful Revascularization for Patients With Coronary Chronic Total Occlusions: A Report of 1,655 Patients

**DOI:** 10.3389/fcvm.2020.00116

**Published:** 2020-07-24

**Authors:** Lei Guo, Xiaoyan Zhang, Haichen Lv, Lei Zhong, Jian Wu, Huaiyu Ding, Jiaying Xu, Xuchen Zhou, Rongchong Huang

**Affiliations:** ^1^Department of Cardiology, The First Affiliated Hospital of Dalian Medical University, Dalian, China; ^2^Department of Radiology, Fuyang Hospital of Anhui Medical University, Fuyang, China; ^3^Department of Cardiology, Capital Medical University Affiliated Beijing Friendship Hospital, Beijing, China

**Keywords:** cardiac mortality, coronary chronic total occlusions, major adverse cardiac event, medical therapy, percutaneous coronary intervention, successful revascularization, outcomes

## Abstract

**Background:** To date, the benefit of successful revascularization of chronic total occlusions (CTOs) on prognosis remains uncertain, and there is a paucity of data on the impact of successful revascularization for CTO patients on long-term cardiovascular survival. This study aimed to investigate the long-term cardiovascular survival for patients with successful and unsuccessful CTO revascularization in a large cohort of patients.

**Methods:** There were 1,655 consecutive patients with at least one CTO included and were grouped into successful revascularization (*n* = 591) and unsuccessful revascularization (*n* = 1,064). Propensity score matching (PSM) was carried out to balance the clinical and the angiographic characteristics. Cardiac mortality was defined as the primary endpoint. Major adverse cardiac event (MACE) was assessed as a “secondary endpoint.”

**Results:** After 3.6 years of follow-up, there was no significant difference between the successful and the unsuccessful revascularization groups in the rate of cardiac mortality [adjusted hazard ratio (HR) 0.96, 95% confidence interval (CI) 0.59–1.58, *p* = 0.865]. After the PSM analysis (371 pairs) between the two groups, the cardiac mortality rate values (HR 0.51, 95% CI 0.23–1.15, *p* = 0.104) were equivalent, whereas the adjusted risk of MACE (HR 0.43, 95% CI 0.32–0.58, *p* = 0.001) and target-vessel revascularization (HR 0.41, 95% CI 0.29–0.58, *p* < 0.001) were significantly higher in patients with unsuccessful revascularization.

**Conclusion:** For the treatment of CTO patients, successful revascularization was not associated with a lesser risk for cardiac mortality as compared with unsuccessful revascularization. However, successful revascularization reduced MACE and target-vessel revascularization.

## Introduction

Coronary chronic total occlusion (CTO) revascularization is a challenging obstacle in the field of coronary intervention (1). Several observational studies demonstrated that better outcomes of successful CTO percutaneous coronary intervention (PCI) included angina reduction and improvement in both long-term survival and left ventricular function in comparison with unsuccessful procedures (2–6). However, CTO-PCI is attempted in 10–20.7% patients (7, 8) mainly because CTO-PCI procedures are costly, complex, and associated with higher complication rates as compared to non-CTO interventions (9, 10). Therefore, a large portion of patients with CTOs are treated by coronary artery bypass grafting (CABG) or medical therapy (MT), especially in patients with well-developed collateral circulation or multiple CTOs and multi-vessel coronary disease (11–15). However, the patients who receive MT without a CTO-PCI attempt were rarely considered previously (16, 17). To date, the prognosis of successful revascularization of CTO remains uncertain, and there is limited data on the impact of successful revascularization for CTO patients on long-term cardiovascular survival.

In this study, we sought to investigate the long-term clinical outcomes of CTO patients treated with successful and unsuccessful CTO revascularization in a large cohort of population.

## Materials and Methods

### Study Population

A total of 1,702 patients with at least one CTO were enrolled at our institution between 2007 and 2016 (18). The exclusion criteria were: (1) prior CABG and (2) acute ST-segment elevation myocardial infarction (MI). After exclusion, a final population of 1,655 patients was left. The subjects were classified into either successful revascularization group or unsuccessful revascularization group based on the initial therapeutic modalities. The patients referred for revascularization had presence of symptomatic angina and/or myocardial viability in the territory of CTO or inducible ischemia, which were reported in our previously published article (18).

Clinical and procedural data and in-hospital outcomes were entered into a dedicated database. Clinical follow-up was performed through examination of hospital records and telephone follow-up or outpatient clinical visit. Our institutional review committee approved the present study in accordance with the Declaration of Helsinki.

### Definitions and Clinical Endpoints

A CTO is defined as a coronary obstruction with thrombolysis in myocardial infarction (TIMI) antegrade grade 0 flow for more than 3 months based on previous coronary angiogram or clinical history (2). Patients who have undergone a successful CTO-PCI were implanted with first-generation drug-eluting stent (DES) from January 2006, including sirolimus-eluting and paclitaxel-eluting stents [Excel, Yinyi, Firebird and Firebird2 (China); Cipher and Taxus (USA)]. The angiographic success of CTO-PCI was defined as <20% residual stenosis and TIMI grade ≥2 flow after the implantation of a DES to the CTO vessel. The extent of collateral circulation flow was assessed according to the validated Rentrop classification scale (19). After PCI, all patients received aspirin indefinitely and clopidogrel for at least 12 months. The “primary efficacy endpoint” was cardiac mortality. A major adverse cardiac event (MACE) was assessed as a “secondary endpoint,” consisting of cardiac mortality, MI, or target-vessel revascularization (TVR). Definitions of cardiac mortality, MI, and TVR have been described in a previous article (18). All patients underwent two-dimensional echocardiography.

### Statistical Analysis

The data are listed as mean ± standard deviation for continuous variables and as percentages for categorical variables. The variables were compared between groups by using Student's *t*-test for continuous variables and chi-square or Fisher exact test for discrete variables. Propensity score matching (PSM) analysis was constructed to adjust for any potential confounder between the two groups based on multivariable logistic regression model. Survival—free of adverse events—was calculated by Kaplan–Meier analysis and compared using log-rank test. In multivariable models, the covariates with *p* < 0.1 on univariate analysis were considered as candidate variables. Cox regression was used to compare adjusted hazard rates based on age, gender, history of MI, chronic kidney disease (CKD), left ventricular ejection fraction (LVEF), taking renin–angiotensin system blockade, left anterior descending coronary artery (LAD) and left circumflex coronary artery (LCX) involvement, multivessel disease, calcification, blunt stump, Japanese-chronic total occlusion (J-CTO) score, and coronary dissection in the total population. In the propensity-matched population, Cox regression was based on blunt stump, bending >45°, J-CTO score, coronary dissection, and perforation. All analyses were carried out with Stata V.15 software (StataCorp, College Station, TX, USA). All tests were conducted at the 0.05 level.

## Results

### Clinical and Angiographic Characteristics

Among the 1,655 patients, of whom 944 were CTOs, 800 patients were treated with medication and 855 were treated with revascularization. In the successful revascularization group, 470 patients underwent successful CTO-PCI and 121 patients got successful CTO CABG. In the unsuccessful revascularization group, 800 patients received MT and 264 patients had a failed PCI. Five CTO-dedicated operators performed the procedures during the study period. A total of 36 patients underwent reattempted CTO-PCI after the prior failed CTO-PCI, and 24 patients got a successful reattempt. The success rate of reattempted CTO-PCI was 66.7%.

Table 1 shows the baseline clinical characteristics of CTO patients between each therapeutic group. As compared to patients who had MT, the patients who had CABG were younger and more often had a familial history of coronary artery disease (CAD). Compared with patients who underwent PCI, the patients who underwent CABG were younger and more frequently had a familial history of CAD and low LVEF. Compared with patients who had MT, those who had PCI were younger and had mostly previously undergone PCI, whereas they less often have a history of MI, with high total cholesterol, high-density lipoprotein cholesterol, and LVEF, but with low low-density lipoprotein cholesterol.

**Table 1:** Baseline clinical characteristics in CTO patients of each therapeutic group.

**Variable**	**MT (A)**	**PCI (B)**	**CABG (C)**	***P*****-value**			
	**(*n* = 800)**	**(*n* = 734)**	**(*n* = 121)**	**Overall**	**B vs. A**	**C vs. A**	**C vs. B**
Age, years	64.8 ± 10.7	63.2 ± 9.7	63.0 ± 9.4	0.001	<0.001	0.046	0.995
Male, %	623 (77.9)	572 (77.9)	97 (80.2)	0.845	0.980	0.570	0.581
Smoking, %	326 (40.8)	309 (42.1)	48 (39.7)	0.809	0.592	0.822	0.616
Hypertension, %	548 (68.5)	482 (65.7)	75 (62.0)	0.480	0.255	0.153	0.431
Diabetes mellitus, %	290 (36.3)	252 (34.3)	50 (41.3)	0.350	0.307	0.281	0.136
Dyslipidemia, %	577 (72.1)	536 (73.0)	97 (80.2)	0.263	0.388	0.104	0.235
TC, (m mol/L)	4.64 ± 1.28	4.50 ± 1.34	4.61 ± 1.30	0.029	0.009	0.672	0.320
TG, (m mol/L)	1.6 (1.1–2.4)	1.5 (1.1–2.4)	1.7 (1.2–2.3)	0.632	0.856	0.363	0.309
HDL-C, (m mol/L)	1.24 ± 0.38	1.29 ± 0.69	1.19 ± 0.33	<0.001	0.049	0.095	0.046
LDL-C, (m mol/L)	2.77 ± 0.87	2.58 ± 0.94	2.75 ± 0.92	<0.001	<0.001	0.944	0.175
Familial history of CAD, %	93 (11.6)	96 (14.1)	28 (23.1)	0.002	0.387	<0.001	0.004
Previous MI, %	406 (50.8)	320 (43.6)	58 (47.9)	0.020	0.005	0.564	0.373
Previous PCI, %	78 (9.8)	105 (14.3)	14 (11.6)	0.023	0.006	0.534	0.421
CKD, %	83 (10.3)	51 (6.9)	8 (6.6)	0.041	0.018	0.196	0.892
LVEF, %	51.7 ± 9.6	54.1 ± 8.1	51.7 ± 9.6	<0.001	<0.001	0.856	0.009

*CABG, coronary artery bypass grafting; CAD, coronary artery disease; CKD, chronic kidney disease; CTO, chronic total occlusion; HDL-C, high-density lipoprotein cholesterol; LDL-C, low-density lipoprotein cholesterol; LVEF, left ventricular ejection fraction; MI, myocardial infarction; MT, medical therapy; PCI, percutaneous coronary intervention; TC, total cholesterol; TG, total triglyceride*.

The characteristics of the enrolled patients are shown in Tables 2, 3. Older patients, previous MI, CKD, and taking renin–angiotensin–aldosterone system blockers were more common among the unsuccessful revascularization group than in the successful revascularization group, whereas high LVEF was more prevalent in the successful revascularization patients. Regarding lesion and procedural characteristics, blunt stump, multivessel disease, LCX involvement, calcification, high J-CTO score, and coronary dissection were more common in the unsuccessful revascularization group, whereas CTO location in LAD was more frequent in the successful revascularization group.

**Table 2:** Baseline clinical characteristics in the successful and the unsuccessful revascularization groups.

	**Total population**			**Propensity-matched population**		
	**Successful revascularization (*n* = 591)**	**Unsuccessful revascularization (*n* = 1,064)**	***P*-value**	**Successful revascularization (*n* = 371)**	**Unsuccessful revascularization (*n* = 371)**	***P*-value**
Age, years	63.0 ± 9.7	64.5 ± 10.4	0.003	63.3 ± 9.3	64.2 ± 10.1	0.280
Male, %	442 (74.8)	850 (79.9)	0.016	281 (75.7)	277 (74.7)	0.734
Smoking, %	239 (40.4)	444 (41.7)	0.610	148 (39.9)	141 (38.0)	0.598
Hypertension, %	388 (65.7)	717 (67.4)	0.473	242 (65.2)	357 (69.3)	0.241
Diabetes mellitus, %	211 (35.7)	381 (35.8)	0.966	135 (36.4)	139 (37.5)	0.761
Dyslipidemia, %	433 (73.3)	777 (73.0)	0.820	280 (75.5)	284 (76.5)	0.731
TC, (m mol/L)	4.54 ± 1.34	4.59 ± 1.28	0.255	4.57 ± 1.36	4.58 ± 1.24	0.831
TG, (m mol/L)	1.61 (1.14–2.41)	1.57 (1.11–2.36)	0.906	1.69 (1.16–2.41)	1.60 (1.09–2.33)	0.532
HDL-C, (m mol/L)	1.30 ± 0.62	1.23 ± 0.49	0.347	1.33 ± 0.69	1.19 ± 0.37	0.207
LDL-C, (m mol/L)	2.62 ± 0.92	2.72 ± 0.90	0.056	2.62 ± 0.91	2.72 ± 0.88	0.322
Familial history of CAD, %	83 (14.0)	134 (12.6)	0.402	48 (12.9)	49 (13.2)	0.913
Previous MI, %	241 (40.8)	543 (51.0)	<0.001	161 (43.4)	169 (45.6)	0.555
Previous PCI, %	77 (13.0)	120 (11.3)	0.292	45 (12.1)	40 (10.8)	0.564
CKD, %	33 (5.6)	109 (10.5)	0.001	22 (5.9)	32 (8.6)	0.158
LVEF, %	53.9 ± 8.4	52.1 ± 9.3	0.002	53.6 ± 8.5	56.6 ± 8.7	0.474
Stable angina, %	148 (25.0)	335 (31.5)	0.006	100 (26.9)	109 (29.4)	0.463
UA/NSTEMI, %	336 (56.8)	486 (45.7)	<0.001	199 (53.6)	178 (48.1)	0.123
**Baseline medication**						
Aspirin, %	578 (97.8)	1,042 (97.9)	0.858	361 (97.3)	365 (98.4)	0.312
Clopidogrel, %	554 (93.7)	969 (91.1)	0.055	346 (93.3)	332 (89.5)	0.067
Statin, %	555 (93.9)	1,008 (94.7)	0.482	350 (94.3)	347 (93.5)	0.644
β blocker, %	438 (74.1)	804 (75.6)	0.513	279 (75.2)	276 (74.4)	0.800
ACEI or ARB, %	341 (57.7)	691 (64.9)	0.004	206 (55.5)	211 (56.9)	0.711

*ACEI, angiotensin-converting enzyme inhibitor; ARB, angiotensin-receptor blocker; CAD, coronary artery disease; CKD, chronic kidney disease; HDL-C, high-density lipoprotein cholesterol; LDL-C, low-density lipoprotein cholesterol; LVEF, left ventricular ejection fraction; MI, myocardial infarction; NSTEMI, non-ST elevation myocardial infarction; PCI, percutaneous coronary intervention; TC, total cholesterol; TG, total triglyceride; UA, unstable angina*.

**Table 3:** Lesion characteristics, procedural characteristics, complications of CTO revascularization, and in-hospital death in the successful and the unsuccessful revascularization groups.

	**Total population**			**Propensity-matched population**		
	**Successful revascularization (*n* = 591)**	**Unsuccessful revascularization (*n* = 1,064)**	***P*-value**	**Successful revascularization (*n* = 371)**	**Unsuccessful revascularization (*n* = 371)**	***P*-value**
One CTO lesion, %	483 (81.7)	902 (84.8)	0.108	303 (81.7)	312 (84.1)	0.380
Two CTO lesions, %	99 (16.8)	152 (14.3)	0.180	61 (16.4)	51 (13.7)	0.305
LAD, %	231 (39.1)	356 (33.5)	0.022	142 (38.3)	151 (40.7)	0.499
LCX, %	144 (24.4)	322 (30.3)	0.011	97 (26.1)	105 (28.3)	0.509
RCA, %	294 (49.7)	517 (48.6)	0.652	184 (49.6)	169 (45.6)	0.270
Multivessel disease, %	447 (75.9)	904 (85.2)	<0.001	332 (81.8)	312 (76.8)	0.083
Proximal or mid, CTO location, %	456 (77.2)	802 (75.4)	0.416	277 (74.7)	275 (74.1)	0.866
Blunt stump, %	237 (40.1)	636 (59.8)	<0.001	149 (40.2)	177 (47.7)	0.038
Calcification, %	103 (17.4)	230 (21.6)	0.042	67 (18.1)	86 (23.2)	0.085
Bending >45°, %	277 (46.9)	545 (51.2)	0.090	161 (43.4)	195 (52.6)	0.012
Length ≥20 mm, %	369 (62.4)	692 (65.0)	0.291	226 (60.9)	231 (62.3)	0.706
Collateral flow ≥ 2^*^	439 (74.2)	817 (76.7)	0.254	271 (73.0)	276 (74.3)	0.739
J-CTO score	1.63 ± 1.15	1.95 ± 1.21	<0.001	1.58 ± 1.16	1.82 ± 1.31	0.014
SYNTAX score	21.7 ± 8.7	23.3 ± 8.9	0.18	22.1 ± 8.6	21.8 ± 8.3	0.724
Myocardial ischemia, %	386 (65.3)	560 (52.6)	<0.001	217 (58.4)	204 (55.0)	0.335
Complete revascularization (except CTO), %	441 (74.6)	762 (71.6)	0.170	279 (75.2)	267 (72.1)	0.318
Number of stents^**^	1.13 ± 1.03	0	–	1.08 ± 1.05	0	–
Stent type^**^						
SES	386 (82.1)	–	–	240 (83.6)	–	–
PES	84 (17.9)	–	–	47 (16.4)	–	–
Coronary dissection, %	0	19 (1.7)	0.001	0	7 (2.1)	0.008
Perforation, %	0	8 (0.7)	0.057	0	4 (1.1)	0.045
In-hospital death, %	2 (0.3)	6 (0.6)	0.719	0	2 (0.5)	0.157

* *Collateral circulation flow (Rentrop grade)*.

** *Stents data were only available for patients who underwent successful CTO-PCI*.

*CTO, chronic total occlusion; J-CTO, Japanese-chronic total occlusion; LAD, left ascending coronary artery; LCX, left circumflex coronary artery; PCI, percutaneous coronary intervention; PES, paclitaxel-eluting stent; RCA, right coronary artery; SES, sirolimus-eluting stent*.

After PSM, 371 pairs of patients were matched. A total of 287 (77.4%) patients had successful CTO-PCI and 84 (22.6%) patients underwent CTO CABG in the successful revascularization group in the propensity-matched population. As for the patients in the unsuccessful revascularization group among the propensity-matched population, 301 (81.1%) patients received MT and 70 (18.9%) patients underwent failed CTO-PCI. The clinical baseline characteristics were not different between the successful and the unsuccessful revascularization groups after PSM.

### Clinical Follow-Up

**Figure 1:**
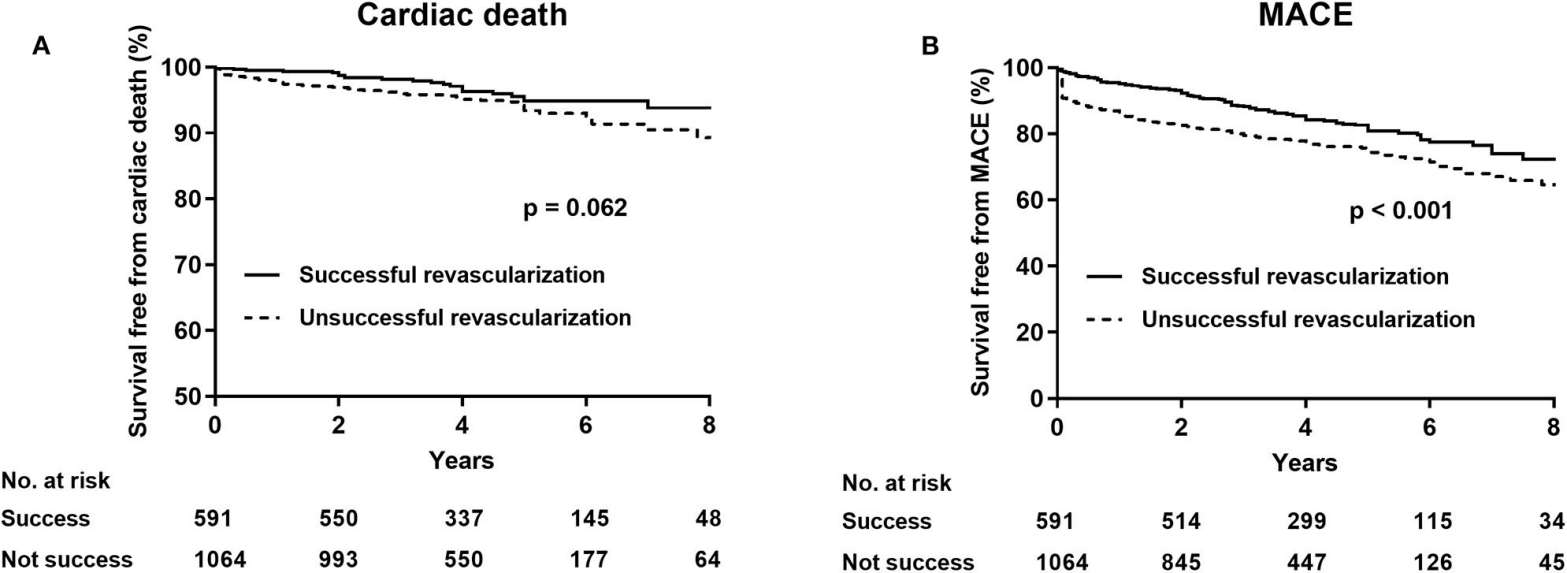
Kaplan–Meier analysis for cardiac death **(A)** and major adverse cardiovascular events **(B)** in the successful revascularization and the unsuccessful revascularization of chronic total occlusion patients.

After a follow-up of 3.6 (interquartile range: 2.1–5.0) years, cardiac death (successful revascularization vs. unsuccessful revascularization: 4.2 vs. 6.0%, unadjusted HR 0.65, 95% CI 0.41–1.03, *p* = 0.065) was similar between the successful and the unsuccessful revascularization groups. After multivariate analyses, no statistically significant difference was observed in terms of cardiac mortality (adjusted HR 0.96, 95% CI 0.59–1.58, *p* = 0.865) and MI (HR 0.88, 95% CI 0.39–2.01, *p* = 0.762), whereas the rate of MACE (HR 0.67, 95% CI 0.53–0.85, *p* = 0.001) and of TVR (HR 0.70, 95% CI 0.52–0.95, *p* = 0.020) were significantly higher in the unsuccessful revascularization group than in the successful revascularization group (Table 4; Figure 1).

**Table 4:** Clinical outcomes in all patients during follow-up.

	**Successful revascularization**	**Unsuccessful revascularization**	**Unadjusted HR (95% CI)**	***P*-value**	**Adjusted HR (95% CI)**	***P*-value**
	**(*n* = 591)**	**(*n* = 1,064)**				
Cardiac death, %	25 (4.2)	64 (6.0)	0.65 (0.41–1.03)	0.065	0.96 (0.59–1.58)	0.865
MI, %	34 (5.8)	78 (7.3)	0.73 (0.49–1.09)	0.126	0.72 (0.47–1.10)	0.127
TVR, %	68 (11.5)	155 (14.6)	0.73 (0.55–0.97)	0.031	0.70 (0.52–0.95)	0.020
MACE, %	105 (17.8)	261 (24.5)	0.65 (0.52–0.81)	<0.001	0.67 (0.53–0.85)	0.001

*CI, confidence interval(s); HR, hazard ratio; MACE, major adverse cardiovascular events; MI, myocardial infarction; TVR, target-vessel revascularization*.

**Figure 2:**
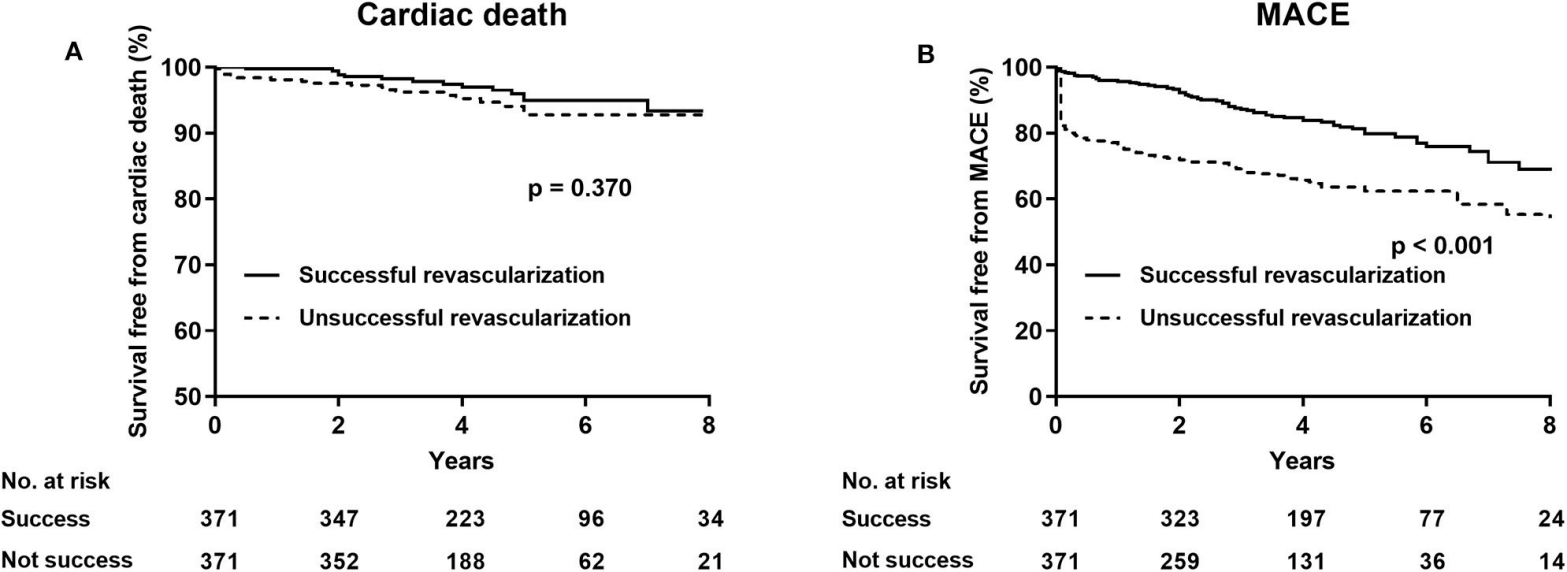
Kaplan–Meier curves for cardiac death **(A)** and major adverse cardiovascular events **(B)** in the successful revascularization and the unsuccessful revascularization of chronic total occlusion patients after propensity score adjustment.

After PSM, the rate of cardiac death (successful revascularization vs. unsuccessful revascularization: 4.6 vs. 5.4%, HR 0.74, 95% CI 0.39–1.43, *p* = 0.347) was not significantly different in the two groups. After multivariate analyses, the rate of cardiac mortality (adjusted HR 0.68, 95% CI 0.36–1.32, *p* = 0.256) and of MI (HR 0.60, 95% CI 0.34–1.06, *p* = 0.080) were comparable, whereas the incidence of MACE (HR 0.43, 95% CI 0.32–0.58, *p* = 0.001) and of TVR (HR 0.41, 95% CI 0.29–0.58, *p* < 0.001) were significantly less in the successful revascularization group than in the unsuccessful revascularization group (Table 5; Figure 2).

**Table 5:** Clinical outcomes in propensity-matched patients during follow-up.

	**Successful revascularization**	**Unsuccessful revascularization**	**Unadjusted HR (95% CI)**	***P*-value**	**Adjusted HR (95% CI)**	***P*-value**
	**(*n* = 371)**	**(*n* = 371)**				
Cardiac death, %	17 (4.6)	20 (5.4)	0.74 (0.39–1.43)	0.374	0.68 (0.36–1.32)	0.256
MI, %	21 (5.7)	30 (8.1)	0.64 (0.37–1.13)	0.123	0.60 (0.34–1.06)	0.080
TVR, %	48 (12.9)	99 (26.7)	0.41 (0.29–0.58)	<0.001	0.41 (0.29–0.58)	<0.001
MACE, %	71 (19.1)	132 (35.6)	0.44 (0.33–0.58)	<0.001	0.43 (0.32–0.58)	0.001

*CI, confidence interval(s); HR, hazard ratio; MACE, major adverse cardiovascular events; MI, myocardial infarction; TVR, target-vessel revascularization*.

### Subgroup Analysis

Since acute ST-segment elevation myocardial infarction (STEMI) was excluded, the severity of unstable angina (UA) or of non-STEMI (NSTEMI) and stable angina are different, based on a subgroup analysis of the CAD subtype of stable angina and UA/ NSTEMI that we have done. No significant interaction was observed between treatment strategy and CAD groups (*p* for interaction = 0.098). The cardiac death rate was comparable for the successful and the unsuccessful revascularization groups between the subgroups analyzed (Table 6).

**Table 6:** Comparative unadjusted hazard ratio of cardiac death for CAD subgroup according to successful and unsuccessful revascularization.

**CAD**	**Number of patients**	**Unadjusted HR (95% CI)**	***P* value**	***P* for interaction**
Stable angina	483	0.58 (0.23–1.48)	0.258	
UA/NSTEMI	822	1.16 (0.63–2.13)	0.618	0.098

*CABG, coronary artery bypass grafting; CAD, coronary artery disease; CI, confidence interval(s); HR, hazard ratio; NSTEMI, non-ST elevation myocardial infarction; UA, unstable angina*.

## Discussion

We assessed the long-term cardiovascular survival of patients with successful and with unsuccessful CTO revascularization in a large cohort of patients and showed that successful CTO revascularization may not reduce cardiac mortality in comparison with unsuccessful revascularization. However, the patients who received unsuccessful revascularization tended to have a higher prevalence of MACE and target-vessel revascularization as compared with patients who underwent successful revascularization.

With advancement in devices and skills, high procedural success rates could be achieved in CTO revascularization (20, 21). However, 44% of all CTO patients received conservative treatment (7). In the DECISION-CTO and EuroCTO trials, PCI was not associated with reducing death, repeated revascularization, and MACE compared to MT (22, 23). It was known that CABG was a common management of CTOs according to guidelines (24), especially in patients with multi-vessel coronary disease (14, 15). However, patients with CTOs who underwent CABG were not included in the two randomized trials.

Previous cohort studies mainly focused on the outcomes of successful PCI, as opposed to a failed procedure among CTO patients, and showed positive outcomes with respect to successful CTO-PCI (4, 25). However, to date, the prognosis of successful revascularization of CTO remains uncertain, and there is limited data on the impact of successful revascularization for CTO patients on long-term cardiovascular survival.

In the present study, patients who had unsuccessful revascularization were older and more likely to have LCX involvement, blunt stump, calcification, and high J-CTO score. A lower LVEF was also more frequently observed among them, which means worse cardiac function. These data were consistent with the finding of Yang and coworkers (26). Therefore, a large proportion of these patients with CTOs was unsuitable for revascularization or was more inclined to fail (27, 28).

To adjust for potential selection bias, we performed the PSM analysis and we found that the cardiac death rates were similar between the groups, concordant with the results of prior studies (29, 30). A prior study revealed that successful revascularization was not associated with reducing cardiac mortality compared to medical therapy (31). In the COURAGE trial comparing PCI with MT in patients with stable coronary heart disease (CHD), PCI did not have priority over medical therapy (32). Similarly, our analysis also demonstrated that successful revascularization did not improve long-term cardiovascular survival. The majority of the enrolled patients in our study were with stable CHD, and the COURAGE trial also included similar patients.

However, we also found that successful revascularization was associated with significantly less target-vessel revascularization and MACE compared with unsuccessful procedures, consistent with prior findings (13, 26, 29). The mechanism of revascularization in reducing the MACEs of CTO patients is unclear, but reducing or eliminating myocardial ischemia may have attributed to the good long-term clinical outcomes (33). The amount of viable myocardium viability in the CTO-related territory can have an influence on LVEF, and successful revascularization was associated with the recovery of hibernating myocardium and reduction in adverse left ventricular remodeling. Depressed LVEF is well-known to be associated with increased risk of MACEs, and the improvement of LVEF increased electrical stability, reduced ventricular arrhythmias (a main reason for cardiac death), and provided the collateral vessels with protection against future adverse events (34).

The difference in survival, free from MACE, dramatically became large in less than several months. We found that TVR was the predominant determinant of MACE. Many patients were with prior coronary stenosis, new lesion, or lesion progression before CTO location and they received, following CABG or repeat PCI for these stenotic lesions, when they had new or persistent angina after optimal medical therapy (13, 29). In addition, stent thrombosis and in-stent restenosis, which are caused by stent under-sizing, presence of residual dissection, and residual disease proximal or distal to the stent lesion, were other reasons for TVR and often appeared in the first few months, especially in first 30 days after PCI (35).

The cost-effectiveness of PCI vs. optimal medical therapy is also an important factor for clinical decision making. It was confirmed that the cost of CTO-PCI was superior to optimal medical therapy (10). A study showed that CTO-PCI satisfies the criteria for cost-effectiveness in a population with chronic stable angina, especially among patients with severe symptoms (10). CTO-PCI could yield a significantly higher cost-effectiveness ratio by reducing adverse outcomes and improving the quality of life as compared to optimal medical therapy. Additionally, reducing potentially preventable complications could reduce the costs of CTO-PCI and enhance the cost-effectiveness of revascularization procedures (36). These results indicate that CTO-PCI might be carefully considered in terms of operative complications, clinical outcomes, and cost-effectiveness.

Limitations should be taken into consideration. Firstly, this was not a randomized–controlled study, even though the potential confounding factors were minimized using PSM. Secondly, the residual ischemia-related CTO after revascularization, during the follow-up of the study patients, was not routinely evaluated by stress echocardiography or SPECT for every CTO patient in the large-sample-size study.

## Conclusions

For treatment of CTO, successful revascularization is not associated with improved long-term cardiovascular survival compared with unsuccessful revascularization. However, successful revascularization is associated with significantly less MACE and target-vessel revascularization. Future randomized studies are warranted to confirm these findings.

## Data Availability Statement

The datasets generated for this study are available on request to the corresponding author.

## Ethics Statement

The studies involving human participants were reviewed and approved by the First Affiliated Hospital of Dalian Medical University. The patients/participants provided their written informed consent to participate in this study.

## Author Contributions

LG prepared the manuscript. All authors contributed to the data collection and analyses, edited the draft manuscript, and approved the final manuscript.

## Conflict of Interest

The authors declare that the research was conducted in the absence of any commercial or financial relationships that could be construed as a potential conflict of interest.
