# Corrigendum: Long-Term Outcomes of Successful Revascularization for Patients With Coronary Chronic Total Occlusions: A Report of 1,655 Patients

**DOI:** 10.3389/fcvm.2021.659972

**Published:** 2021-02-15

**Authors:** Lei Guo, Xiaoyan Zhang, Haichen Lv, Lei Zhong, Jian Wu, Huaiyu Ding, Jiaying Xu, Xuchen Zhou, Rongchong Huang

**Affiliations:** ^1^Department of Cardiology, The First Affiliated Hospital of Dalian Medical University, Dalian, China; ^2^Department of Radiology, Fuyang Hospital of Anhui Medical University, Fuyang, China; ^3^Department of Cardiology, Capital Medical University Affiliated Beijing Friendship Hospital, Beijing, China

**Keywords:** cardiac mortality, coronary chronic total occlusions, major adverse cardiac event, medical therapy, percutaneous coronary intervention, successful revascularization, outcomes

In the published article, there was an error in the author affiliations. Instead of “**Rongchong Huang**^**3**^,” it should be “**Rongchong Huang**^**1,3**^.”

The authors apologize for this error and state that this does not change the scientific conclusions of the article in any way. The original article has been updated.

